# Nitro-Oleic Acid-Mediated Nitroalkylation Modulates the Antioxidant Function of Cytosolic Peroxiredoxin Tsa1 during Heat Stress in *Saccharomyces cerevisiae*

**DOI:** 10.3390/antiox11050972

**Published:** 2022-05-14

**Authors:** Lorena Aranda-Caño, Raquel Valderrama, José Rafael Pedrajas, Juan C. Begara-Morales, Mounira Chaki, María N. Padilla, Manuel Melguizo, Francisco Javier López-Jaramillo, Juan B. Barroso

**Affiliations:** 1 Group of Biochemistry and Cell Signaling in Nitric Oxide, Department of Experimental Biology, Faculty of Experimental Sciences, University Institute of Research in Olive Groves and Olive Oils, Campus Las Lagunillas, University of Jaén, E-23071 Jaén, Spain; laranda@ujaen.es (L.A.-C.); ravalde@ujaen.es (R.V.); pedrajas@ujaen.es (J.R.P.); jbegara@ujaen.es (J.C.B.-M.); mounira@ujaen.es (M.C.); npadilla@ujaen.es (M.N.P.); 2 Department of Inorganic and Organic Chemistry, Faculty of Experimental Sciences, Campus Las Lagunillas, University of Jaén, E-23071 Jaén, Spain; mmelgui@ujaen.es; 3 Institute of Biotechnology, University of Granada, E-18071 Granada, Spain; fjljara@ugr.es

**Keywords:** peroxiredoxin, Tsa1, nitro-oleic acid, nitroalkylation, heat stress, nitro-fatty acids, *Saccharomyces cerevisiae*

## Abstract

Heat stress is one of the abiotic stresses that leads to oxidative stress. To protect themselves, yeast cells activate the antioxidant response, in which cytosolic peroxiredoxin Tsa1 plays an important role in hydrogen peroxide removal. Concomitantly, the activation of the heat shock response (HSR) is also triggered. Nitro-fatty acids are signaling molecules generated by the interaction of reactive nitrogen species with unsaturated fatty acids. These molecules have been detected in animals and plants. They exert their signaling function mainly through a post-translational modification called nitroalkylation. In addition, these molecules are closely related to the induction of the HSR. In this work, the endogenous presence of nitro-oleic acid (NO_2_-OA) in *Saccharomyces cerevisiae* is identified for the first time by LC-MS/MS. Both hydrogen peroxide levels and Tsa1 activity increased after heat stress with no change in protein content. The nitroalkylation of recombinant Tsa1 with NO_2_-OA was also observed. It is important to point out that cysteine 47 (peroxidatic) and cysteine 171 (resolving) are the main residues responsible for protein activity. Moreover, the in vivo nitroalkylation of Tsa1 peroxidatic cysteine disappeared during heat stress as the hydrogen peroxide generated in this situation caused the rupture of the NO_2_-OA binding to the protein and, thus, restored Tsa1 activity. Finally, the amino acid targets susceptible to nitroalkylation and the modulatory effect of this PTM on the enzymatic activity of Tsa1 are also shown in vitro and in vivo. This mechanism of response was faster than that involving the induction of genes and the synthesis of new proteins and could be considered as a key element in the fine-tuning regulation of defence mechanisms against oxidative stress in yeast.

## 1. Introduction

One of the main stresses that affects yeast cells is the variation in growth temperature. Specifically, when growth temperature changes from 30 °C to 37 °C, a classic heat stress is triggered [1]. One of the main consequences of heat stress is the overproduction of reactive oxygen species (ROS), which generate lipid peroxidation, protein oxidation and genetic damage through DNA modification, which in turn, imply damage to a wide range of cellular components. However, to restore the redox balance, a protective transcriptional program called the heat shock response (HSR) and the set of antioxidant systems are activated to prevent cell death and to enable cells to cope with exposure to high growth temperatures [2,3].

The antioxidant response occurs by non-enzymatic (i.e., ascorbic acid and glutathione (GSH)) and enzymatic defenses, such as catalase (CAT), superoxide dismutases (SODs), methionine sulfoxide reductase (MSR), thioredoxins (Trx) and peroxiredoxins (Prx). Prx, also called thioredoxin peroxidases (Tpx) or “protective proteins”, are important conserved proteins involved in antioxidant defense and redox signaling because they perform antioxidant and chaperone functions and are able to regulate signal transduction cascades [4,5]. This protective activity is carried out by the active cysteine residues responsible for the reduction in peroxides. Depending on the number of cysteine residues implicated in the catalysis mechanism, Prx are divided in two categories: 1-Cys (Tpx mitochondrial) and 2-Cys. In the latter group, an additional division gives rise to two classes: atypical (peroxisomal Ahp1 and nuclear Tpx) and typical 2-Cys Prx (cytoplasmic Tsa1 and Tsa2) [4,6].

The catalytic mechanism for Prx consists of three phases: peroxidation, resolution and recycling. The peroxidation step is common to the three groups. The conserved peroxidatic cysteine (C_P_) thiol is oxidized to sulfenic acid (SOH) by the peroxide substrate. In 2-Cys Prx, the resolution stage generates the formation of a disulfide bond between the C_P_ and the thiol group of another cysteine (resolving, C_R_). Within the atypical 2-Cys Prx, both C_P_ and C_R_ are contained in the same polypeptide because they are monomeric. Nevertheless, typical 2-Cys Prx are constituted by identical homodimers where the interacting C_R_ and C_P_ are located in different subunits [4]. For 1-Cys Prx, the resolution process is carried out by other reducing agents, such as glutathione, lipoic acid and cyclophilin [7,8]. Finally, in the recycling stage, the regeneration of free thiols (C_P_ and C_R_) by thioredoxins (Trx) or any similar molecule to Trx takes place [4,9].

The HSR has been previously described to also be induced by new signaling molecules called nitrated fatty acids (NO_2_-FAs), also known as nitroalkenes or nitrolipids, which are generated by the non-enzymatic interaction of reactive nitrogen species (RNS), such as nitric oxide (NO) and nitrogen dioxide (NO_2_), with unsaturated fatty acids [10,11]. These NO_2_-FAs have been identified in a wide range of plant and animal species under both physiological and stress conditions. Given their amphipathic nature, NO_2_-FAs can be stabilized in micelles or liposomes in aqueous solutions. The formation of amphipathic structures reduces their reactivity with nucleophilic molecules [12,13]. Although the knowledge about the interaction mechanism for NO_2_-FAs with biomembranes is still limited, NO_2_-FAs have been shown to affect the structural dynamics of integral cell membrane proteins [14].

NO_2_-FAs can also establish adducts with proteins. The electrophilic nature of NO_2_-FAs allows them to carry out reversible Michael additions with protein nucleophiles such as cysteine (Cys), histidine (His) and lysine (Lys) residues to generate a post-translational modification (PTM) called nitroalkylation. This PTM entails a reversible change in protein structure and function, and represents the main mechanism by which NO_2_-FAs perform their biological function [11,15,16,17].

Adduction with proteins is considered one of the stores of NO_2_-FAs, together with esterification in complex lipid structures [13,18]. NO_2_-FAs can be released from their reservoirs recovering their electrophilic signaling potential because NO_2_-FAs adducts are reversible and, otherwise, esterified forms can be hydrolyzed by esterases and lipases [10].

In animal systems, NO_2_-FAs have shown therapeutic benefits because of their powerful anti-inflammatory and cytoprotective effects in various experimental models [19,20,21,22,23,24]. For instance, the treatment of endothelial cells with nitro-oleic acid (NO_2_-OA) directly activates the HSR. The elicitation of HSR is an emerging therapy against diseases that affect protein folding and conformation. Thus, NO_2_-OA represents a new kind of HSR inducer [25].

In plant systems, NO_2_-FAs have become signaling molecules during plant development and under abiotic stress conditions (salinity, heavy metals, mechanical stress and low temperature) through the induction of heat shock transcription factors that regulate the expression of antioxidant systems [26] or trigger ROS production [27]. The ability of nitro-linolenic acid (NO_2_-Ln) to release NO has recently been shown. This capacity enables the in vivo generation of S-nitrosoglutathione (GSNO) and directly points out the contribution of NO_2_-FAs to S-nitrosothiols (SNO) homeostasis in plant cells [28].

Given the relevant participation of NO_2_-FAs in the activation of the HSR, as well as their demonstrated implication in the response to stress in animal and plant systems, the main objective of this article focuses on determining the role of NO_2_-FAs in the modulation of Tsa1 antioxidant activity during heat stress. This work specifically demonstrates the NO_2_-OA mediated nitroalkylation of the antioxidant protein Tsa1 during heat stress in *Saccharomyces cerevisiae* as the experimental model. We also identify the endogenous presence of this nitrated lipid in yeast for the first time. Finally, the amino acid targets susceptible to nitroalkylation and the negative effect of this PTM on the enzymatic activity of Tsa1 are also shown.

## 2. Materials and Methods

### 2.1. Organisms, Liquid Medium and Growth Conditions

Wild-type *Saccharomyces cerevisiae* strains (BY4741: MATa; his3D1; leu2D0; met15D0; ura3D0) were obtained from EUROSCARF. Yeast cells were grown at 30 °C with shaking in a medium (YPD) containing 1% (*w*/*v*) yeast extract.

### 2.2. Heat Stress Conditions

Yeast cultures were kept in YPD medium under the growth conditions until an A_600 nm_ = 0.6 was achieved. In this stage, a group of control cells were collected and another group of cells were subjected to heat stress (37 °C) for 1 h.

Cells (control and heat stress) were collected by centrifugation at 3000× *g* and 4 °C for 5 min. The cell pellet was washed with cold distilled water and subjected to the same centrifugation conditions twice.

### 2.3. Obtaining the Cell-Free Extract

Yeast cells were lysed by sonication for 1 h at 4 °C in a homogenization buffer (ratio 1:5 (*w*/*v*)) containing 50 mM Tris-HCl, pH 7.5, 0.1 mM DTT, 0.1% (*v*/*v*) Triton X-100, 1 mM EDTA, 1 mM PMSF, 10% (*v*/*v*) commercial cocktail of protease inhibitors (AEBSF, 1,10-phenanthroline, pepstatin A, leupeptin, bestatin and E-64; Sigma-Aldrich. St. Louis, MO, USA) and 5 mg of Zymolyase 20T (USBiological) per gram of cells. Sonicated cells were then centrifuged at 10,000× *g* and 4 °C for 30 min. The supernatant that constituted the cell-free extract (CFE) was subjected to different analyses.

### 2.4. Quantification of Hydrogen Peroxide (H_2_O_2_)

H_2_O_2_ was measured following the method described by Bellincampi et al. [29] with some modifications. The presence of reductants in the CFE was initially removed by using desalting columns (GE Healthcare. Buckinghamshire, UK). A volume of 500 μL of free-reductants CFE was incubated for 45 min in the dark with the same volume of the assay reagent: 500 μM (NH_4_)_2_Fe(SO_4_)_2_·6H_2_O, 50 mM H_2_SO_4_, 200 μM xylenol orange and 200 mM sorbitol. The H_2_O_2_-mediated oxidation of Fe^2+^ to Fe^3+^ was determined spectrophotometrically by measuring the absorbance at 560 nm of the Fe^3+^-xylenol orange complex. A calibration curve with a H_2_O_2_ standard solution was also constructed. All the experiments were performed in triplicate. The H_2_O_2_ values were expressed as nmoles of H_2_O_2_ per gram of dry weight.

### 2.5. Enzymatic Peroxiredoxin Activity Assay

Peroxiredoxin activity was carried out in a reaction mixture containing 50 mM HEPES buffer, pH 7.0, 250 µM NADPH, 0.5 µM Trr2p (Thioredoxin reductase 2p), 5 µM Trx3p, peroxiredoxin Tsa 1 and 100 µM peroxide. Before adding peroxide, the reaction mixture was incubated at 25 °C for 5 min. The disappearance of NADPH at 340 nm was spectrophotometrically monitored for 10 min.

The peroxide used to determine recombinant Tsa1 enzymatic activity was H_2_O_2_ instead of t-BuOOH, which was the substrate for the in vivo determination of Tsa1 activity in cell extracts because this peroxide is only reduced by peroxiredoxins, while H_2_O_2_ can be a substrate of catalase and peroxiredoxins. All the experiments were performed in triplicate. Activity values were expressed as mU per mg of protein.

### 2.6. Immunodetection of Tsa1

20 µg of protein from the heat stress and control CFE samples were separated by 10% SDS-PAGE and transferred to PVDF membranes (Immobilon P, Millipore, Bedford, MA, USA) for immunodetection. A specific antibody against Tsa1 of *Saccharomyces cerevisiae* (Santa Cruz Biotechnology, Dallas, TX, USA) was used at a dilution of 1:500. The immunoreactive band was detected by chemiluminescence using photographic films (Hyperfilm, Amersham Pharmacia Biotech. Buckinghamshire, UK) and luminol.

### 2.7. Synthesis and Characterization of the NO_2_-OA Standard and the ^13^C18-NO_2_-OA Internal Standard by NMR Spectroscopy

NO_2_-OA synthesis was carried out by nitroselenation, oxidation and hydroselenoxide elimination in a similar way to that previously described in the work of Baker et al. [30]. Thus, commercial oleic acid (OA) (1 g, 3.05 mmol), solid mercury chloride (1.15 g, 4.23 mmol), phenylselenyl bromide (0.93 g, 3.93 mmol) and sodium nitrite (0.49 g, 7.19 mmol) were dissolved in a tetrahydrofuran–acetonitrile mixture (1:1, *v*/*v*, 24 mL) in Ar atmosphere for 12 h. After incubation, solids were removed by filtration and the solvent was evaporated. The residue was dissolved in 12 mL of tetrahydrofuran. Additionally, 33% (*v*/*v*) hydrogen peroxide (3.63 mL) was added dropwise and the mixture was shaken for 1 h in an ice bath. Then, an extraction with hexane was carried out. Afterward, the solvent was washed with a saturated sodium chloride solution, dried with anhydrous magnesium sulfate, filtered and evaporated. The dry residue was obtained in a hexane/diethyl ether/acetic acid mixture (5 mL, 80:20:0.5, *v*/*v*/*v*) and decontaminated by flash column chromatography (silica gel 60) with a mixture of hexane/diethyl ether/acetic acid (80:20:0.5, *v*/*v*/*v*). The fractions of interest were selected by TLC plates (particle size 25 mm, 0.2 mm thick, Fluka Alu sheets) developed with a mixture of hexane/diethyl ether/ acetic acid (80:20:0.5, *v*/*v*/*v*). TLCs were revealed with both UV light (the nitro group absorbs UV light) and iodine (which reacts with the double bonds of the molecule). In this way, the fractions enriched in NO_2_-OA, but free of oleic acid, were selected. Finally, its structure was analyzed by NMR with a Bruker Avance 400 spectrometer (Billerica, MA, USA) operating at 400.13 MHz for 1 h and 100.61 MHz for 13C. Spectra processing and calculations were performed with the ACD/Labs software, version 12.01, 2009 (Advanced Chemistry Development, Toronto, ON, Canada). Carbon 13-labeled NO_2_-OA (^13^C18-NO_2_-OA), which was used as internal standard in the NO_2_-OA quantification protocol, was synthesized as described above.

### 2.8. Lipid Extraction and Acid Hydrolysis

Lipids present in the control and heat stress samples were extracted by the Bligh and Dyer method [31]. Next, the chromatographic protocol described by Fazzari et al. [18] was followed. The chromatography fractions were evaporated, resuspended in 50 µL of methanol and mixed with 10 nM ^13^C18-NO_2_-OA as internal standard to quantify the loss during the acidic hydrolysis process. To limit the artificial acid-catalyzed nitration reactions, 250 µL of methanolic sulfanilamide (1 g/10 mL) were added to the sample and incubated for 20 min. Later, the sample was evaporated and incubated in 2.5 mL of acetonitrile/hydrochloric acid (9:1) for 1 h at 90 °C. Following acid hydrolysis, NO_2_-FAs were extracted with hexane/H_2_O (2:1). Finally, the hexane fraction was isolated, evaporated and resuspended in methanol to be analyzed by LC-MS/MS.

### 2.9. Detection and Identification of Endogenous NO_2_-OA in Saccharomyces Cerevisiae

The detection, identification and quantification of the NO_2_-OA levels in yeast samples were performed using a triple quadrupole linear trap mass spectrometer (QTRAP 6500^+^, SCIEX) coupled with an ultra-high-performance liquid chromatograph (UHPLC) ExionLC AD of the same brand. Lipid extracts were separated by means of a Kinetex 1.7 µm C18 100 Å column (150 × 2.1 mm) in a solvent system composed of A (0.1% (*v*/*v*) formic acid) and B (methanol with 0.1% (*v*/*v*) formic acid) with the following gradient program: 1st) starts at 10% B and rises to 95% B in 5 min; 2nd) isocratic gradient with 95% B from 5 to 10 min; 3rd) after 10.1 min, it returned to the initial conditions and stayed the same for 5 min to re-equilibrate the column. The flow rate was 0.3 mL/min.

The MS/MS analysis was performed in the negative ion mode using oscillating collision. The desolvation temperature was 350 °C. The presence of NO_2_-OA was confirmed by multiple reaction monitoring (MRM) scanning with specific transitions for NO_2_-OA (326.1/46 *m*/*z*) and ^13^C18-NO_2_-OA (344.1/46 *m*/*z*).

The quantification of the NO_2_-OA levels was performed using a calibration curve with NO_2_-OA standard concentrations ranging from 0.5 nM to 18.75 nM containing 10 nM ^13^C18-NO_2_-OA as internal standard to correct the loss caused by both the processing method and the detection technique. The Sciex OS software was used. All the experiments were carried out in triplicate.

### 2.10. Expression and Purification of Recombinant Tsa1

Plasmid pHTP1, containing the Tsa1 gene, was transformed into *E. coli* BL21 cells. The transformed cells were cultured at 37 °C in LB medium supplemented with kanamycin (50 µg/mL) until the early exponential growth phase was reached (A_600 nm_ = 0.4–0.6). Recombinant protein production occurred after additional incubation at 16 °C for 16 h. The labeling of the protein with a 6-His tag allowed its purification by immobilized metal affinity chromatography (IMAC). The purified Tsa1 was analyzed by 14% SDS-PAGE and stained with Coomassie blue dye. Although the theoretical molecular mass was 21.6 kDa, the real molecular mass of recombinant Tsa1 was 24.5 kDa because the His-tag was linked to a Tobacco Etch Virus (TEV) protease recognition sequence at the N-terminal end (Supplementary Figure S1).

### 2.11. In Vitro Nitroalkylation of Recombinant Tsa1

Before carrying out the nitroalkylation treatment, recombinant Tsa1 was incubated for 30 min with shaking and TCEP (tris(2-carboxyethyl)phosphine) in 50 mM buffer Hepes at 1:20 concentration ratio (protein: TCEP) to reduce the disulfur bridges between cysteines inside the protein. Next, the presence of reductants in the sample was eliminated by using Spin desalting columns (Thermo Scientific, Rockford, IL, USA).

For the nitroalkylation treatment, recombinant Tsa1 was incubated for 30 min at 37 °C with shaking and NO_2_-OA at 1:10 concentration ratio (protein: NO_2_-OA). As negative controls, the recombinant protein was treated under the same conditions with either methanol (the solvent of NO_2_-OA) or the non-nitrated form of NO_2_-OA, oleic acid (OA, ratio 1:10).

To check the absence of NO release by NO_2_-OA during nitroalkylation treatment, an aliquot of recombinant Tsa1 was incubated for 30 min at 37 °C with shaking and 200 µM of the NO scavenger cPTIO (2-4-carboxyphenyl-4,4,5,5-tetramethylmidazoline-1oxyl-3-oxide) before the NO_2_-OA treatment.

The statistical significance between means was analyzed by the Student’s *t*-test.

### 2.12. Extraction of the In Vivo Nitroalkylated Peptides

The CFEs of the control and heat stress samples were subjected to 70% acetone precipitation for 12 h at −20 °C. Next, samples were centrifuged at 10,000× *g* and 4 °C for 15 min; the pellet containing the precipitated proteins was reserved. Samples were solubilized in 50 mM ammonium bicarbonate, pH 8.0 before performing a proteolytic digestion with chymotrypsin (1:20; CFE protein concentration: protease) for 12 h at 37 °C with shaking. Once the protein peptides were obtained, samples were enriched in nitroalkylated peptides with a diethyl ether extraction (1:1, *v*/*v*). The non-polar upper and interface phases (enriched in nitroalkylated peptides) were isolated, evaporated and resuspended in 0.1% (*v*/*v*) formic acid reaching a concentration of 0.1 µg peptides/µL. After being filtered through 0.2 µm filters, samples were analyzed by nano-LC-MS/MS.

### 2.13. Detection of Protein Nitroalkylation by nano-LC-MS/MS

Nitroalkylation detection was carried out by mass spectrometry. Nano-liquid chromatography (Nano-LC) was performed on an EASY-nLC 1000 (Thermo Scientific) with an Easy-Spray RSLC C18 2 µm column (50 cm × 75 µm; Thermo Scientific). Previously, the peptide mixture was loaded in a Pepmap100 C18 3 µm Nanoviper 2P precolumn (75 µm × 2 cm; Thermo Scientific) for 5 min at a flow rate of 5 μL/min. Peptide separation was performed at 40 °C for all the assays. Mobile phase buffer A and mobile phase B were composed of water and 0.1% (*v*/*v*) formic acid and acetonitrile and 0.1% (*v*/*v*) formic acid, respectively. Samples were separated at 200 nL/min by 162-min chromatography. Mobile phase B increased from 8% to 65% over a 130-min period, and 65% to 100% over a 1-min period, followed by a 5-min wash with 100% B and 26-min re-equilibration with 2% B. After elution, peptide cations were converted into ions in the gas phase by nano-electrospray ionization (ESI) and analyzed on a Thermo Orbitrap Q-Exactive (Thermo Scientific). The mass spectrometer operated in the positive mode to perform the full scan mode. For the MS1 mass analysis, the search for precursor peptides was performed within the 300–1500 *m*/*z* range with a resolution of 70,000, an automatic gain control (AGC) of 1e6 and a maximum injection time of 50 ms. The MS2 tandem mass analysis established a range of peptides between 200 and 2000 *m*/*z* with a resolution of 17,500, an automatic gain control (AGC) of 2e5 and a maximum injection time of 80 ms.

### 2.14. MS/MS Data Processing and Identification of Nitroalkylated Proteins

The identification of the protein sequences from the mass spectrometry data was carried out using the Proteome Discoverer 1.4 software (Thermo Scientific) with the SEQUEST HT search engine and the UniProt *Saccharomyces cerevisiae* database. The modifications established during the search were: cysteine carbamidomethylation (+57,021 Da) as fixed modification; methionine oxidation (+15,995 Da) and nitroalkylation (+327,241 Da) as dynamic modifications. Chymotrypsin was fixed as the proteolytic enzyme and up to four missing cleavage sites were allowed. The mass tolerances for the parent and fragment ions were set at 10 ppm and 0.02 Da. The statistical validation of these data was obtained by the Percolator tool with a 1% FDR (false discovery rate). Finally, the proteins with at least three unique identified peptides were retained in this study.

### 2.15. Relative Quantification of Tsa1 Nitroalkylation by nano-LC-MS/MS

The relative quantification of the in vitro nitroalkylation of Tsa1 was carried out by a directed search of the peptides of interest. To develop this method, information on the spectrometric behavior of the peptides of interest was required. This was why the NO_2_-OA-nitroalkylated recombinant Tsa1 was used as a tool to define the m/z search windows of the precursor ions and retention times (RTs) (Table S1).

Next, the directed search consisted of selecting the peptides, which contain those susceptible to nitroalkylation targets in both their modified and unmodified forms to quantify the number of peptide spectrum matches (PSM) of each one in order to perform a relative quantification oriented to identify those most susceptible to nitroalkylation residue in the recombinant protein (Supplementary Table S1).

The detection and relative quantification of the in vivo nitroalkylated residues were performed using the Xcalibur program (Thermo Fisher Scientific). The spectrometric parameters (*m*/*z* and RT) of the peptides/precursor ions extracted from the NO_2_-OA-nitroalkylated recombinant Tsa1 were used as nitroalkylation standards. Subsequently, the bioinformatic search in the MS1 of the control and stress samples was conducted considering the m/z of the precursor ion with a mass tolerance of 25 ppm and ±0.2 min in the RT (Supplementary Table S2).

### 2.16. Docking

Docking of the model of *Saccharomyces cerevisiae* Tsa1 with NO_2_-OA was carried out at the SwissDoc sever in accurate mode and without defining the region of interest (blind docking) but allowing flexibility for the side chains within 5 Å of any atom of the ligand in its reference binding mode [32,33]. Results were analyzed with UCSF Chimera [34].

The coordinates of the NO_2_-OA were obtained from the ZINC database [35] (entry 43021575) and those of the protein were generated from the structure of the C47S mutant (PDB entry 3sbc) [36] after truncation to include only four subunits and regeneration of the coordinates of C47.

## 3. Results

### 3.1. Heat Stress Activates Tsa1 Enzymatic Activity

**Figure 1:**
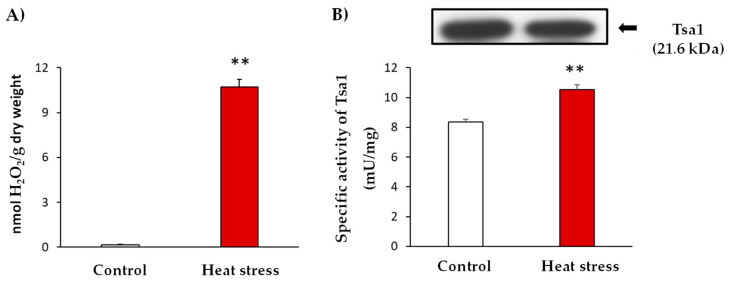
Characterization of heat stress at 37 °C for 1 h in yeast cultures in the exponential growth phase (A_600 nm_ = 0.6). (**A**). Quantification of H_2_O_2_ levels in the control and stress situations expressed as nmol H_2_O_2_/g dry weight. (**B**) Specific activity and Tsa1 protein expression by immuno-blot. The control and heat stress samples (20 µg protein per well) were subjected to SDS-PAGE. Proteins were electroblotted on PVDF membranes and then incubated with anti-Tsa1 antibody (1:500). The results are the mean ± SEM of at least three independent experiments. Statistical significance between means were analyzed by Student’s *t*-test and differences were significant when *p* < 0.001 (**).

Similar to other stress situations, heat stress triggers oxidative damage. In line with this, yeast culture cells were subjected to heat stress and the occurrence of oxidative stress was evidenced by the determination of hydrogen peroxide (H_2_O_2_) levels. These analyses showed an up to 59-fold increase in H_2_O_2_ levels after the heat stress (Figure 1: Panel A). In addition, the enzymatic activity of Tsa1 was approximately 30% higher in the stress situation (Figure 1: Panel B). Interestingly, heat stress generated an increase in the activity which was not caused by a protein expression induction (Figure 1: Panel B). This finding suggests a post-translational modification (PTM) of this protein in response to the generated stress.

### 3.2. Detection and Quantification of Endogenous NO_2_-OA Levels

**Figure 2:**
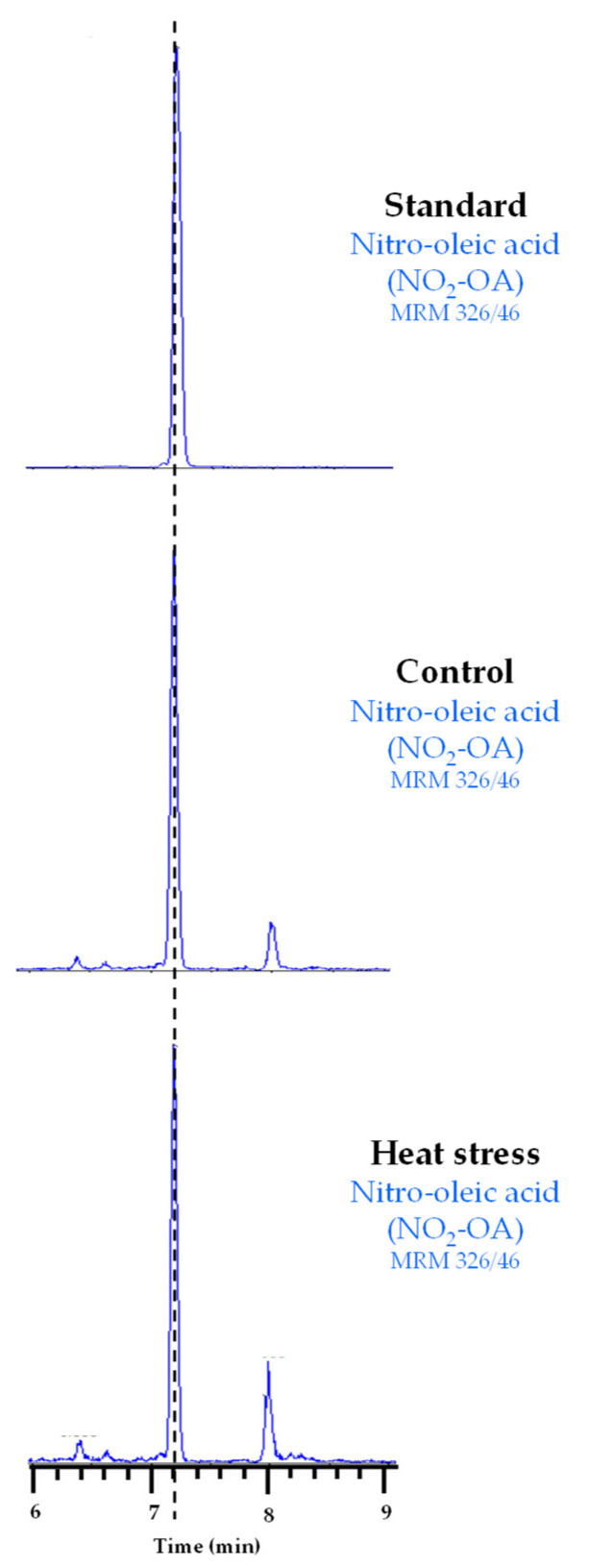
Chromatographic peaks corresponding to the NO_2_-OA standard and its endogenous detection in control and heat stress yeast samples. The dotted vertical line indicates the peaks with the same retention time.

Our results suggest that Tsa1 activity is regulated by a PTM under heat stress conditions. In line with this, NO_2_-FAs are signaling molecules closely related to heat stress because they have been identified as inducers of genes related to the HSR in both animal and plant models [25,26]. NO_2_-FAs are also able to regulate the protein function by PTMs [16,17]. Therefore, we explored whether the regulation of Tsa1 activity during heat stress could be mediated by these NO_2_-FA-dependent PTMs. First, the endogenous presence of NO_2_-FAs in yeast had to be identified because the physiological occurrence of NO_2_-FAs has only been reported in animals and plants to date. To do so, the lipid extraction of the control and heat stress samples was analyzed by LC-MS/MS by monitoring the 326/46 MRM transition (*m/z*). This corresponds to NO_2_-OA because the full mass ion spectra (MS) of the NO_2_-OA standard showed a major ion product with *m/z* of 326 when analyzed in the negative ion mode and the MS/MS (MS_2_) spectra showed a major fragment with *m/z* of 46 corresponding to the NO_2_ molecule. Our results show, in both control and heat stress yeast samples, a chromatographic peak coinciding with the 326/46 MRM transition and the RT of the NO_2_-OA standard (Figure 2), which confirmed the endogenous occurrence of this NO_2_-FA in yeast for the first time.

**Figure 3:**
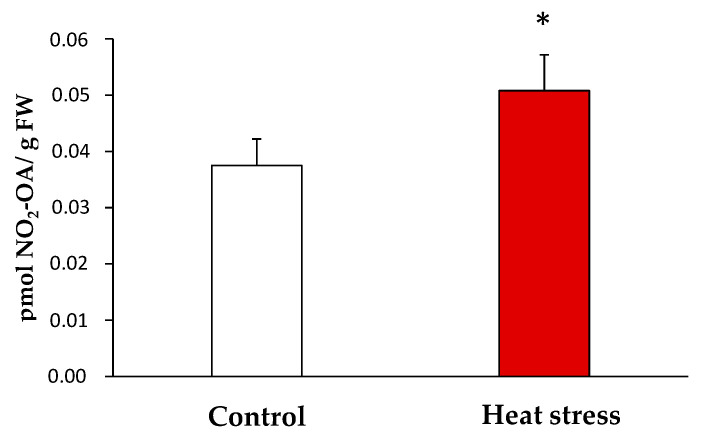
Endogenous levels of NO_2_-OA detected by LC-MS/MS in yeast heat stress and control samples. NO_2_-OA abundance is expressed as pmol NO_2_-OA per gram of fresh weight. The results are the mean ± SEM of at least three independent experiments. Statistical significance between means were analyzed by Student’s *t*-test and differences were significant when *p* < 0.05 (*). FW: fresh weight.

Once the endogenous presence of NO_2_-OA in the yeast samples was detected, the quantification of its levels under the control and stress conditions was carried out by LC-MS/MS. In the control, the NO_2_-OA levels were 0.038 ± 0.01 pmol/g fresh weight (FW) while 0.051 ± 0.01 pmol NO_2_-OA /g FW were detected in the heat stress situation. Therefore, the heat stress situation brought a significant increase (approximately 35%) in the NO_2_-OA levels *versus* the control situation (Figure 3).

### 3.3. Effect of NO_2_-OA on the Enzymatic Activity of Tsa1

NO_2_-FAs can modulate the enzymatic activity by a PTM called nitroalkylation, which involves its adduction with Cys, Lys or His residues. Otherwise, they can act as NO donors and this NO can, in turn, mediate the S-nitrosylation of proteins [16,37]. In order to clarify the effect of NO_2_-OA on the enzymatic activity of Tsa1, the recombinant protein was incubated with NO_2_-OA (ratio 1:10; protein: NO_2_-OA). As negative controls, recombinant Tsa1 treated with methanol (solvent of NO_2_-OA; ratio 1:10) or oleic acid (OA, the non-nitrated fatty acid; ratio 1:10) was also tested.

**Figure 4:**
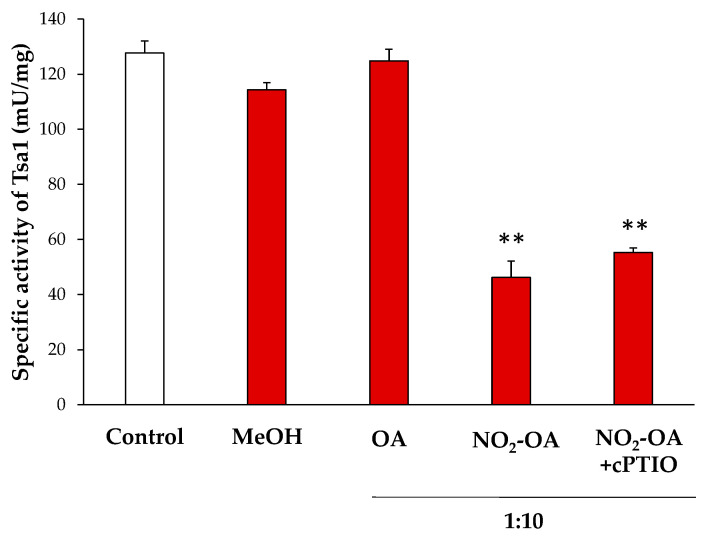
Effect of the NO_2_-OA treatment on the enzymatic activity of peroxiredoxin Tsa1. The specific activity (mU/mg prot) in presence of methanol (MeOH, the NO_2_-OA solvent), oleic acid (OA, the non-nitrated form of NO_2_-OA), and NO_2_-OA, both without and with the cPTIO pretreatment (NO scavenger), is shown. The concentration ratio was 1:10 (protein concentration: NO_2_-OA or OA). The results are the mean ± SEM of at least three independent experiments. Statistical significance between means were analyzed by Student’s *t*-test and double asterisk (**) indicates significant differences (*p* < 0.001) from the control.

The treatment of the recombinant protein with NO_2_-OA led to an approximate 70% decrease in enzymatic activity. In contrast, methanol and OA did not significantly change the specific activity. To rule out the decrease in the activity by the S-nitrosylation derived from the NO release by NO_2_-OA, recombinant Tsa1 was incubated along with a NO scavenger (cPTIO) and NO_2_-OA. In this case, no differences in relation to the NO_2_-OA treatment were observed. Therefore, the S-nitrosylation modification of the protein was not responsible for the regulation of the Tsa1 activity (Figure 4).

### 3.4. Characterization of the Nitroalkylation of Recombinant Tsa1 from Yeast

**Figure 5:**
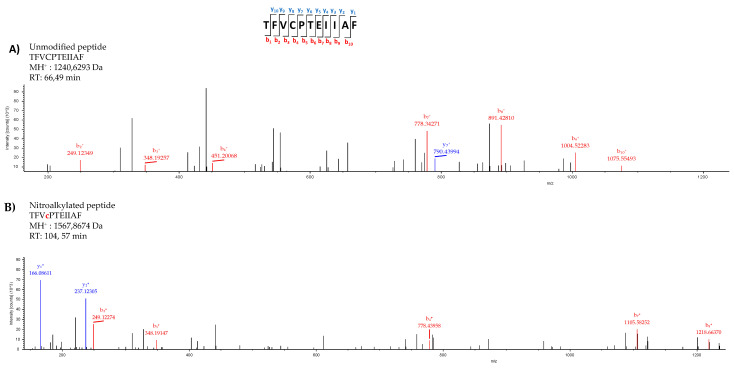
Comparison of the unmodified (**A**) and nitroalkylated (**B**) MS/MS spectra of one of the peptides identified after treating the recombinant Tsa1 with NO_2_-OA. Fragmentation of the precursor ion of the peptide TFVCPTEIIAF generated a series of peptide fragments called either “b” if the charge was retained at the N-terminus or “y” if the charge remained at the C-terminus. The subscripts indicates the charge of ions.

After excluding the negative modulation of Tsa1 by S-nitrosylation, nitroalkylation emerged as the main PTM responsible for this behavior. This hypothesis was reinforced by the fact that the primary structure of Tsa1 contained nitroalkylation targets given the presence of nucleophilic amino acids, such as Cys, His and Lys, susceptible to adduction with NO_2_-OA. The nano LC-MS/MS analysis of the recombinant Tsa1 treated with NO_2_-OA showed the identification of Tsa1 with 100% coverage. The MS/MS spectra of the unmodified peptide TFVCPTEIIAF (MH^+^:1240.6293) closely matched the MS/MS fragmentation pattern of the nitroalkylated peptide (MH^+^:1567.8674). However, an increase in mass in the b_4_ fragmented ion (778.4395 Da) was present only in the nitroalkylated peptide, but not in the b_4_ fragmented ion (451.2006 Da) of the unmodified peptide. This increase corresponded to the NO_2_-OA (327 Da) molecular weight and confirmed the binding of NO_2_-OA to a cysteine residue. The linking of NO_2_-OA in the nitroalkylated peptide made it more non-polar and, consequently, its RT increases to 104.57 min (RT of the unmodified peptide = 66.49 min) (Figure 5).

The above-described behavior was also detected in the other peptides that appear in Table 1. These analyses allowed cysteines 47 and 171 and histidines 105 and 136 as preferential targets of nitroalkylation by NO_2_-OA in Tsa1.

### 3.5. Relative Quantification of the Recombinant Tsa1 Nitroalkylation Targets

**Figure 6:**
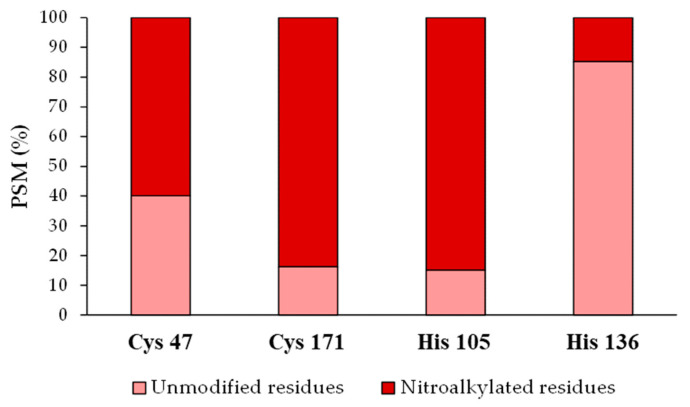
Relative quantification of the recombinant Tsa1 nitroalkylated residues after the treatment with NO_2_-OA. This figure shows the relative percentage (%) of the PSM identified for Cys 47, Cys 171, His 105 and His 136 residues in either the modified or the unmodified form. PSM: number of peptide spectrum matches. His: histidine, Cys: cysteine.

After identifying the nitroalkylation targets in the NO_2_-OA-treated recombinant Tsa1, a relative quantification of the nitroalkylated residues was carried out. This process was achieved by taking into account the number of PSM detected after performing a directed search of the nitroalkylated and unmodified peptides in the mass spectrometer. Cysteine 171 and histidine 105 were nitroalkylated to a greater extent, followed by cysteine 47 and histidine 136 (Figure 6).

### 3.6. Analysis of the Target Residues Involved in Tsa1 Nitroalkylation

**Figure 7:**
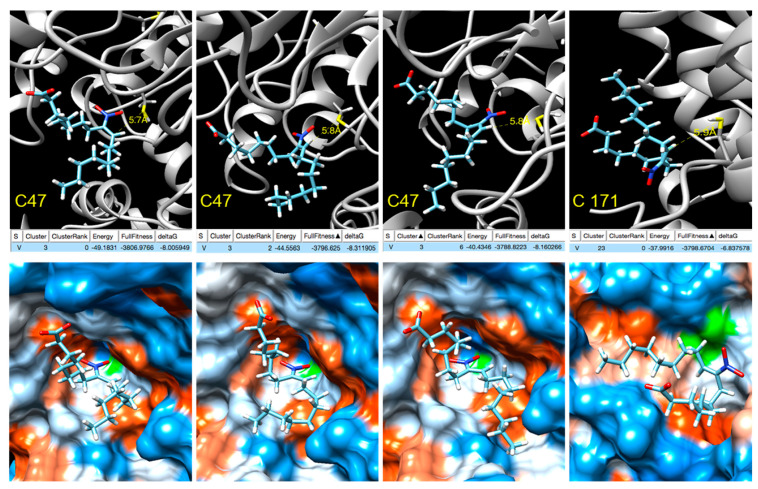
NO_2_-OA docking with Tsa1 Cys 47/Cys 171 (truncated). Active site. Top: Docking poses where the β-carbon next to the nitro group is within 6 Å from the sulfur atoms of the peroxidatic Cys 47 and resolving Cys 171 showing the energy, fullfitness scoring and computed ΔG values. Bottom: Hydrophobic surface colored by the amino acid hydrophobicity on the Kyte–Doolittle scale, from dodger blue for the most hydrophilic to white to orange red for the most hydrophobic.

In order to gain insight into the modulation of Tsa1 by NO_2_-OA, blind docking studies were carried out on the truncated form of the Tsa1 [(α_2_)_5_] complex (PDB entry 3SBC) [36] Tsa1 [(α_2_)_2_], consisting of two consecutive dimers representative of both the dimeric form and the complex (Supplementary Figure S2). A total of 253 ranked docking poses with fullfitness scoring [38] ranging from −3809 kcal/mol to −3780 kcal/mol were analyzed and NO_2_-OA was found to be docked at a few locations of the Tsa1 [(α_2_)_2_], none were close to His residues (Supplementary Figure S3). Several docking poses were near cysteine residues and a closer inspection revealed that in four of them, the distance between the sulfur atom of Cys 47 was within 6 Å from the β-carbon next to the nitro group, supporting the nucleophilic attack to yield the nitroalkylation of the cysteine. Three of these docking poses correspond to cluster 3 and show the interaction with Cys 47, with computed ΔG between −8.0 and −8.3 kcal/mol, while the fourth, with computed ΔG of −6.8 kcal/mol, shows the docking to Cys 171 (Figure 7).

### 3.7. Detection of the In Vivo Nitroalkylation of Tsa1 in the Control and Stress Situations

Having evidenced the negative effect of NO_2_-OA-mediated nitroalkylation on the enzymatic activity of recombinant Tsa1, along with the endogenous detection of NO_2_-OA in yeast, the in vivo modulation of Tsa1 by nitroalkylation and the role of this PTM during heat stress were determined. For this purpose, the nitroalkylation targets previously identified and characterized in the treated recombinant Tsa1 were sought in control and heat stress yeast samples (Supplementary Table S2 and Supplementary Figures S4 and S5). A directed bioinformatic search of the spectra of precursor ions (MS1) from nitroalkylated peptides was carried out.

After this search, only the presence of peptide AFIPLAFTFVcPTEIIAF, which contained nitroalkylated cysteine 47, was confirmed in the control samples (Supplementary Figure S6). However, no other nitroalkylated peptide identified in recombinant Tsa1 was found in the in vivo experiment (Table 2).

## 4. Discussion

One of the most stressful situations in yeast cells is the disturbance in growth temperature. Yeasts specifically exhibit optimal growth at 30 °C, but when temperature rises to 37 °C, a heat stress situation is generated in yeasts [39]. One of the consequences of such stress is a higher reactive oxygen species (ROS) production that could lead to oxidative stress [3,40,41]. In this regard, the growth of yeast cells at 37 °C for 1 h increased both the H_2_O_2_ levels by 59-fold and the cytosolic peroxiredoxin Tsa1 activity, an antioxidant protein responsible for detoxifying ROS implicated in the restoring of the intracellular redox state [42,43]. Interestingly, this significant increase observed in yeast Tsa1 activity did not correlate with a rise in protein expression levels, which suggests a post-translational modification of Tsa1 in the *Saccharomycces cerevisiae* HSR.

The activation of the HSR counteracts the negative effects caused by heat stress in cells. The HSR induces the expression of heat shock proteins (HSPs), most of which are molecular chaperones that help to prevent or reverse protein misfolding [44,45]. In this sense, Tsa1 has been suggested as a chaperone during both oxidative stress and heat stress situations [6,42].

At this point, we hypothesized that nitrofatty acids (NO_2_-FAs) could be responsible for the post-translational modification of Tsa1 because these new signaling molecules have been related to the activation of the HSR and the chaperone network [25,26,46], which suggests a key role for these molecules in the heat stress response. NO_2_-FAs are pleiotropic signaling molecules that trigger a powerful antioxidant response. These molecules are generated by the interaction of RNS, such as nitric oxide (NO) and peroxynitrite (ONOO^-^), with unsaturated fatty acids [12,19,26]. Interestingly, nitro-oleic acid (NO_2_-OA) and conjugated nitro-linoleic acid (NO_2_-cLA) have been described as potent inducers of the HSR in human endothelial cells, where the anti-inflammatory and antioxidant functions of NO_2_-FA-regulated genes have been described [25]. A similar behavior has been observed in *Arabidopsis thaliana* cell cultures treated with nitro-linolenic acid (NO_2_-Ln), where a large set of NO_2_-Ln induced genes were related to the HSR family genes and the antioxidant response [26]. To date, the endogenous presence of nitro-oleic acid (NO_2_-OA) and nitro-linolenic acid has been established in plants [26,47]. Additionally, NO_2_-OA, nitro-linoleic acid (NO_2_-LA), conjugated nitro-linoleic acid and nitro-arachidonic acid (NO_2_-AA) have been identified in animals [48,49,50]. However, the presence of these molecules in *Saccharomyces cerevisiae* had not been reported before the present study, where the presence of NO_2_-OA is shown for the first time. In yeast, only monounsaturated fatty acids such as oleic acid (OA, 18:1) and palmitoleic acid (PA, 16:1) appear because only the Δ9-desaturase enzyme is present. This enzyme catalyzes the insertion of a double bond at the 9-cis position in saturated fatty acids to yield its monounsaturated derivative [51]. Therefore, in yeast, only OA or PA would be available to react with RNS to generate the corresponding NO_2_-FAs. We searched nitrated oleic acid because this molecule had been detected in other species, and its implications in cell signaling processes had been previously described [52,53,54,55,56]. In line with this, the NO_2_-OA levels in *S. cerevisiae* in the non-stress situation were 0.038 ± 0.01 pmoles/g FW, which fall within the range with the concentration of other signaling molecules.

NO_2_-FAs exert their signaling function mainly through the post-translational modification (PTM) by nitroalkylation of Cys, His and Lys residues of proteins. Although information on this PTM is scarce, some proteins have been identified as targets of nitroalkylation in animals and plants, which highlights the regulation of the antioxidant enzyme ascorbate peroxidase (APX) by NO_2_-Ln in Arabidopsis plants [11]. On the other hand, NO_2_-FA are also NO-donors, and the released NO can, in turn, modify proteins by S-nitrosylation [13,37]. After establishing the heat stress-dependent rise in Tsa1 enzymatic activity (not related to a rise in protein expression levels) and bearing in mind our pioneer detection of the endogenous presence of NO_2_-OA in yeasts, the characterization of the effect of NO_2_-OA on the recombinant Tsa1 protein was addressed. Our results demonstrated that the NO_2_-OA treatment decreased recombinant Tsa1 enzymatic activity. This effect could initially be a consequence of nitroalkylation or S-nitrosylation. The implication of S-nitrosylation was ruled out by the pretreatment of recombinant Tsa1 with a NO scavenger (cPTIO), which did not cause the recovery of the enzymatic activity and, therefore, suggested that this PTM could not be responsible for Tsa1 regulation after the treatment with NO_2_-OA. Consequently, we investigated whether the nitroalkylation mediated by NO_2_-OA regulated Tsa1 activity. In the nano-LC-MS/MS analysis of the recombinant Tsa1 incubated with NO_2_-OA, a series of nitroalkylated peptides whose molecular mass increased by 327 Da, which corresponded to the NO_2_-OA molecule, were identified. Nitroalkylated Cys 47, Cys 171, His 105 and His 136 were identified. It is worth highlighting the nitroalkylation of Cys 47 (peroxidatic) and 171 (resolving) for their catalytic implication in the Tsa1 function [57].

The nitroalkylation mechanism, from a chemical point of view, consists of a Michael addition between the protein, that acts as a nucleophile, and an activated olefin as a nitroalkene, where the presence of a nitro-group activates the β-carbon of the double bond. The reactivity of the proteins is determined by the presence of the nucleophilic groups in the side chain of cysteine (i.e., thiol), lysine (i.e., amine) and histidine (i.e., alkylimidazole) [58]. Whereas pH is not a limiting factor in the nucleophilicity of thiols, cysteine is the most reactive residue. However, the reactivity of histidine and lysine, with pKa values of 6.5–7.0 and 9.5–10.0, respectively, is conditioned by the pH of the medium, which determines their degree of protonation (i.e., reduction of nucleophilicity). The reactivity of arginine at physiological pH, with a pKa value > 12, may be residual. Hence, residues prone to nitroalkylation are cysteine, histidine and, to a lesser extent, lysine.

Furthermore, nitroalkylation of proteins as a post-translational modification is a selective process in terms of both target protein and target residue and it cannot rely solely on reactivity. Interactions between the target protein and the nitro-fatty acid play a major role in bringing the reacting groups together, increasing the relative concentration and promoting the reaction. Many docking poses are located at the cavity where the catalytic cysteine residues are placed and, in some of them, the β-carbon of NO_2_-OA is within 6 Å from the sulfur atom (Figure 7). A closer inspection reveals that three poses correspond to the interaction of the NO_2_-FA with the peroxidatic Cys 47, while the fourth shows the docking to the resolving Cys 171 (Figure 7). These results support the feasibility for nitroalkylation of the catalytic cysteine residues as post-translational modification and are in agreement with the results of the nitroalkylation of Tsa1 with NO_2_-OA in vitro.

From the docking poses, computed values of ΔG can be calculated and Kd values estimated by applying the expression ΔG = RTlnKd. Although these values may differ considerably from the experimental dissociation constant, they provide a first approach to the relative affinity. Based on these computed values (Figure 7), NO_2_-OA is expected to have higher affinity for the peroxidatic cysteine, and this prediction is reasonable when considering that it is generally accepted that the peroxidatic cysteine is the stronger nucleophile that attacks and reduces the peroxide substrate.

These facts influence and interfere with Tsa1 catalytic activity because the binding of NO_2_-OA to the peroxidatic cysteine may prevent the binding of the peroxide substrate, and the binding of NO_2_-OA to the resolving cysteine may prevent its binding to the peroxidatic cysteine of another Tsa1 protein. Therefore, the NO_2_-OA-mediated nitroalkylation of Tsa1 on the two Cys residues (peroxidatic and resolving) would not affect a single Tsa1 protein because the resolving Cys performed its action on the peroxidatic cysteine of another molecule of Tsa1. In short, the nitroalkylation of peroxidatic and resolving cysteines was the reason for the drop in Tsa1 enzymatic activity. It is noteworthy that peroxidatic Cys was conserved in the peroxiredoxin family. Therefore, the susceptibility to nitroalkylation of other components in this family became patent.

Given the difficulties to identify the nitroalkylated target proteins and their modified amino acid residues in vivo, in control and heat shock stress yeast samples, we performed a directed search by nano-LC-MS/MS for the nitroalkylated peptides previously identified in recombinant Tsa1. In the control situation, the nitroalkylation of peroxidatic Cys 47 was detected. However, in stress samples, no nitroalkylated peptide was found (Table 2). In addition, this finding is reinforced by the fact that the analysis of the docking poses reveals that NO_2_-OA interacts with Tsa1 at few areas, and none are close to histidine residues, suggesting that in vivo cysteine residues are the target.

Furthermore, in the heat stress situation, a rise in endogenous NO_2_-OA levels (0.051 ± 0.01 pmol/g FW), *versus* the control (0.038 ± 0.01 pmol/g FW), were observed. Nitroalkylation is a reversible post-translational modification that can act as a selective signaling pathway in stressful environments. Thus, in situations accompanied by a rise in ROS and RNS levels, as heat stress, the stability of the nitroalkylation link can be compromised. Indeed, it is well-established that ROS/RNS can cause the oxidation of the bond between the nucleophilic residue and the electrophilic NO_2_-FA (Michael adduct), which leads to the cleavage of the link. This process results in the nitroalkene releasing, which allows the protein to recover its functional activity [19,59,60], as shown in Figure 1: Panel B.

It is also interesting to note that the 2-Cys Prx in yeast can act as chaperones. Accordingly, oxidative stress triggers the switch from low-molecular-weight Prx to a high molecular weight that act as chaperones in response to the generated stress. These changes appear to be mediated by Cys 47, which could act as a sensor of H_2_O_2_ in cells [42]. At this point, the binding of NO_2_-OA to Cys 47 could prevent Tsa1 oligomerization under physiological conditions. However, once the oxidative stress has been generated, the release of this NO_2_-OA could facilitate Cys 47 oxidation and, therefore, the formation of the high-molecular-weight Tsa1 that acts as a chaperone to protect yeast against heat shock stress [42].

The complexity involved in detecting a PTM as nitroalkylation must be highlighted. To date, this modification had only been identified and characterized in in vitro studies [61]. This makes the present research work the first study to detect the in vivo NO_2_-OA-mediated nitroalkylation.

## 5. Conclusions

**Figure 8:**
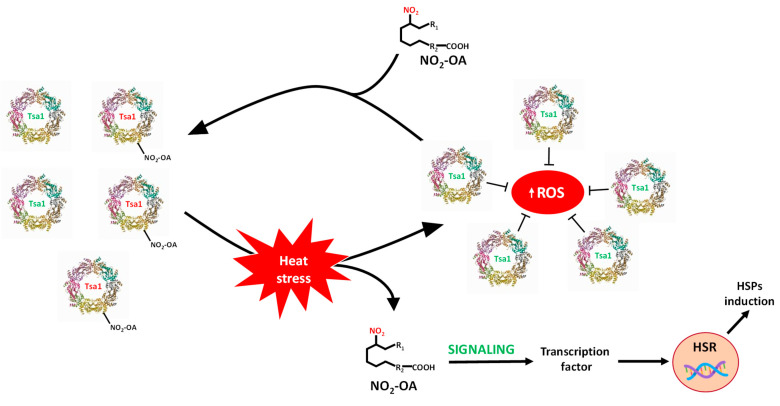
Signaling mechanism by Tsa1 nitroalkylation. In a physiological situation, there is a set of inactivated Tsa1 proteins by the binding of NO_2_-OA to the catalytic residue (peroxidatic Cys). Heat stress generates an oxidative stress situation where ROS levels increase. The rise in ROS content causes the cleavage of the adduct between NO_2_-OA and Tsa1, which leads to both the release of NO_2_-OA and the gain in Tsa1 activity. Tsa1 antioxidant activity would participate in the neutralization of ROS. Additionally, free NO_2_-OA molecules can, in turn, participate in the activation of heat shock response (HSR) genes. HSPs: heat shock protein; ROS: reactive oxygen species.

This study shows the ubiquitous and extensive occurrence of NO_2_-FAs because they are present in animals, plants and yeasts. It also highlights the signaling role of these molecules in the stress response of yeast by means of the nitroalkylation of the Tsa1 enzyme. To summarize, this work provides new insights into the regulation of *Saccharomyces cerevisiae* Tsa1 by the PTM called nitroalkylation. It is important to note that this PTM had not been previously studied in yeast cells. In the control situation, a set of endogenous Tsa1 proteins might be inactivated by NO_2_-OA-mediated nitroalkylation of peroxidatic cysteine. The oxidative stress that derived from heat stress injury brought a rise in ROS levels. To counteract its toxic effects, the concomitant activation of the HSR genes and antioxidant proteins, such as Tsa1, occurs. It is noteworthy that the increase in Tsa1 activity was not a consequence of the rise in its expression levels. Indeed, the restoration of Tsa1 activity was due to the ROS-dependent cleavage of the Michael adduct between peroxidatic Cys 47 sulfide and NO_2_-OA. This response mechanism was faster than that involving the induction of genes and the synthesis of new proteins and could be considered as a key element in the fine-tuning regulation of defense mechanisms against oxidative stress in yeast. Furthermore, in the heat stress situation, an increase in free NO_2_-OA levels also occurred. These nitroalkene molecules, which came mainly from the scission of the adduction with the Tsa1 protein, could participate in its time as inducers of HSR genes (Figure 8).

## Figures and Tables

**Table 1:** Detection by nano-LC-MS/MS of the nitroalkylated peptides of the recombinant Tsa1 treated with NO_2_-OA and the identification of the target residues after the adduction with NO_2_-OA. The position of the nitroalkylation target is highlighted in lowercase. Cys: cysteine; His: histidine.

Recombinant Tsa1 Nitroalkylated Peptides	Nitroalkylation Target
TFVcPTEIIAF	Cys 47
AFIPLAFTFVcPTEIIAF	
AFTFVcPTEIIAF	
TDKNGTVLPcNW	Cys 171
TDKNGTVLPcNWTPGAATIKPTVEDSKEY	
QWTDKNGTVLPcNW	
LADTNhSLSRDY	His 105
ADTNhSLSRDY	
LADTNhSL	
IIDPKGVIRhITINDLPVGRNVDEAL	His 136

**Table 2:** The in vivo identification of the nitroalkylation targets in yeast Tsa1. The m/z and retention time (RT) parameters of the peptide containing nitroalkylated Cys 47 in both in vitro sample (recombinant Tsa1 treated with NO_2_-OA) and control and heat stress yeast samples are shown. RT: retention time; ND: not detected.

Nitroalkylated Target	Nitroalkylation Standard: Recombinant Tsa1 Treated with NO_2_-OA			Endogenous Nitroalkylation (Control)			Endogenous Nitroalkylation (Heat Stress)		
	Nitroalkylated Peptide	*m*/*z*	RT	*m*/*z*	RT	Intensity	*m*/*z*	RT	Intensity
**Cys 47**	AFIPLAFTFVcPTEIIAF	1164.65	117.63	1164.62	117.7	1.58 × 10^4^	ND		

## Data Availability

Data is contained within the article and supplementary material.

## Supplementary Materials

The following supporting information can be downloaded at: https://www.mdpi.com/article/10.3390/antiox11050972/s1.
