# Supplementary material for: Nitro-Oleic Acid-Mediated Nitroalkylation Modulates the Antioxidant Function of Cytosolic Peroxiredoxin Tsa1 during Heat Stress in Saccharomyces cerevisiae
Source: Antioxidants (Basel). 2022 May 14;11(5):972. doi: 10.3390/antiox11050972 (PMC9137801; doi:10.3390/antiox11050972)
Supplement: Supplementary file 1 [file antioxidants-11-00972-s001.zip › antioxidants-1637006-supplementary.pdf]

A)

MGSSHHHHHHSSGPQQGLRENLYFQGMVA
QVQKQAPTFKKTAVVDGVFDEVSLDKYKGK
YVVLAFIPLAFTFVCPTEIIAFSEAAKKFEEQG
AQVLFASTDSEYSLLAWTNIPRKEGGLGPINI
PLLADTNHSLSRDYGVLIEEEGVALRGLFIID
PK
GVIRHITINDLPVGRNVDEALRLVEAFQWTD
KNGTVLPCNWTPGAATIKPTVEDSKEYFEA
ANK

B)

kDa **1** **2**

96

66

48

40

32

26 ◄ **Tsa 1 (24,5 kDa)**

Supplementary Figure S1. Analysis of recombinant Tsa1 from *Saccharomyces cerevisiae*. A) Sequence of the recombinant Tsa1. The underlined area represents the His-tag followed by a Tobacco Etch Virus (TEV) protease recognition sequence. B) SDS-PAGE showing the molecular weight of recombinant Tsa1. Line 1: molecular weight markers. Line 2: purified protein.

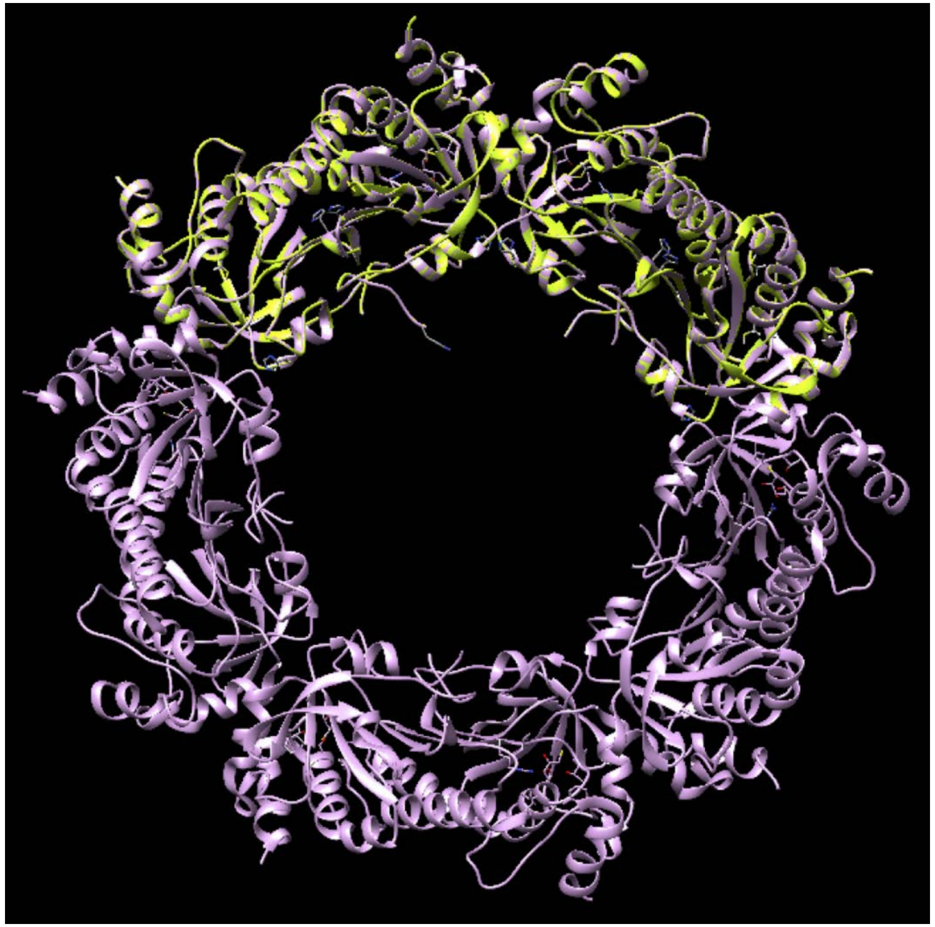

Supplementary Figure S2. Structure of the *S. cerevisiae* Tsa1 $[(\alpha_2)_5]$ complex (PDB entry 3SBC) (bright ube) and of the truncated form $[(\alpha_2)_2]$ (yellow), from [36].

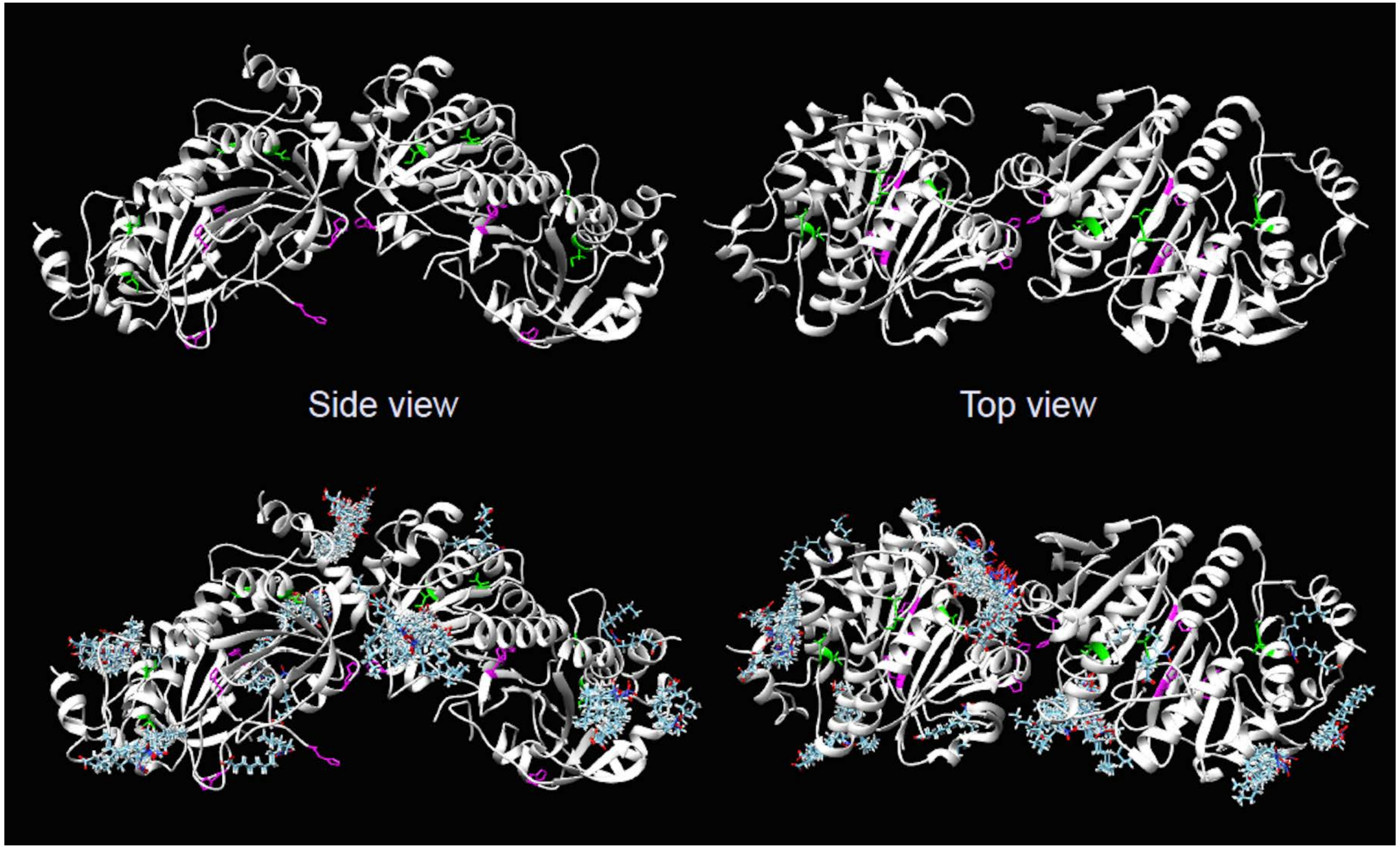

Supplementary Figure S3. Structure of the truncated Tsa1 [$(\alpha_2)_2$] from two views (top figures) and mapping of the NO_2-OA docking (bottom figures).

A)

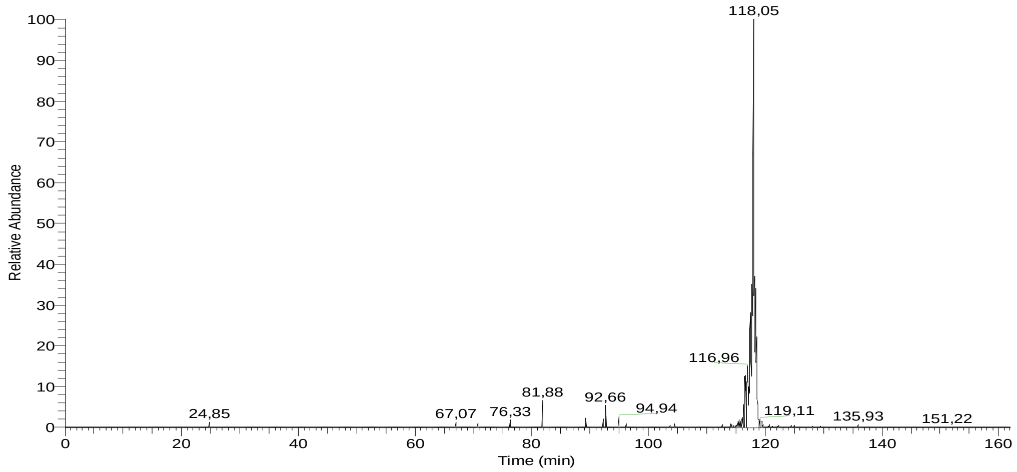

B)

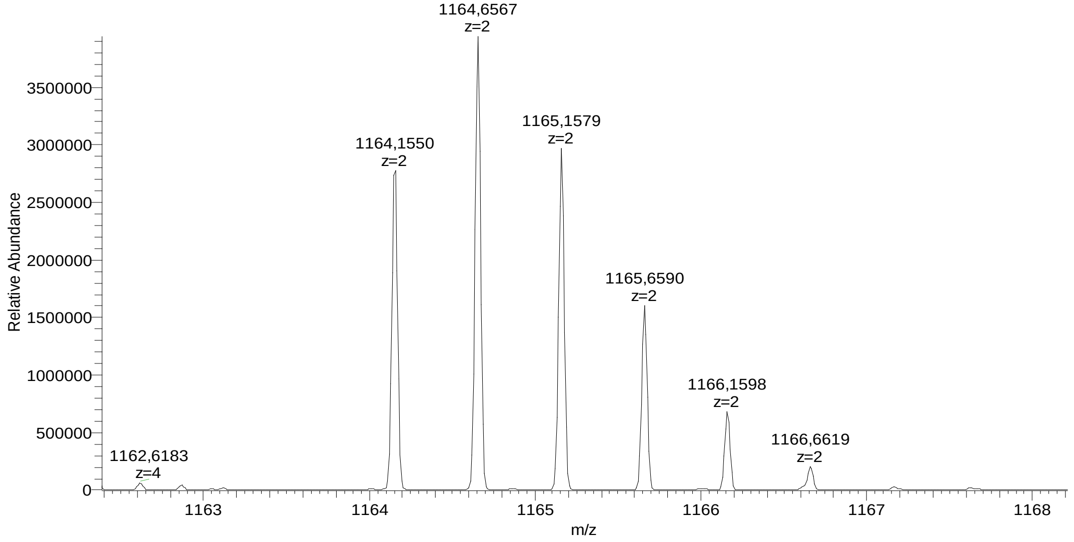

Supplementary Figure S4. Extracted ion chromatogram (A) and MS1 mass spectrum (B) of the peptide AFIPLAFTFVcPTEIIAF (m/z= 1164,65) which contains nitroalkylated cysteine 47 from the recombinant Tsa1 protein treated with NO_2-OA.

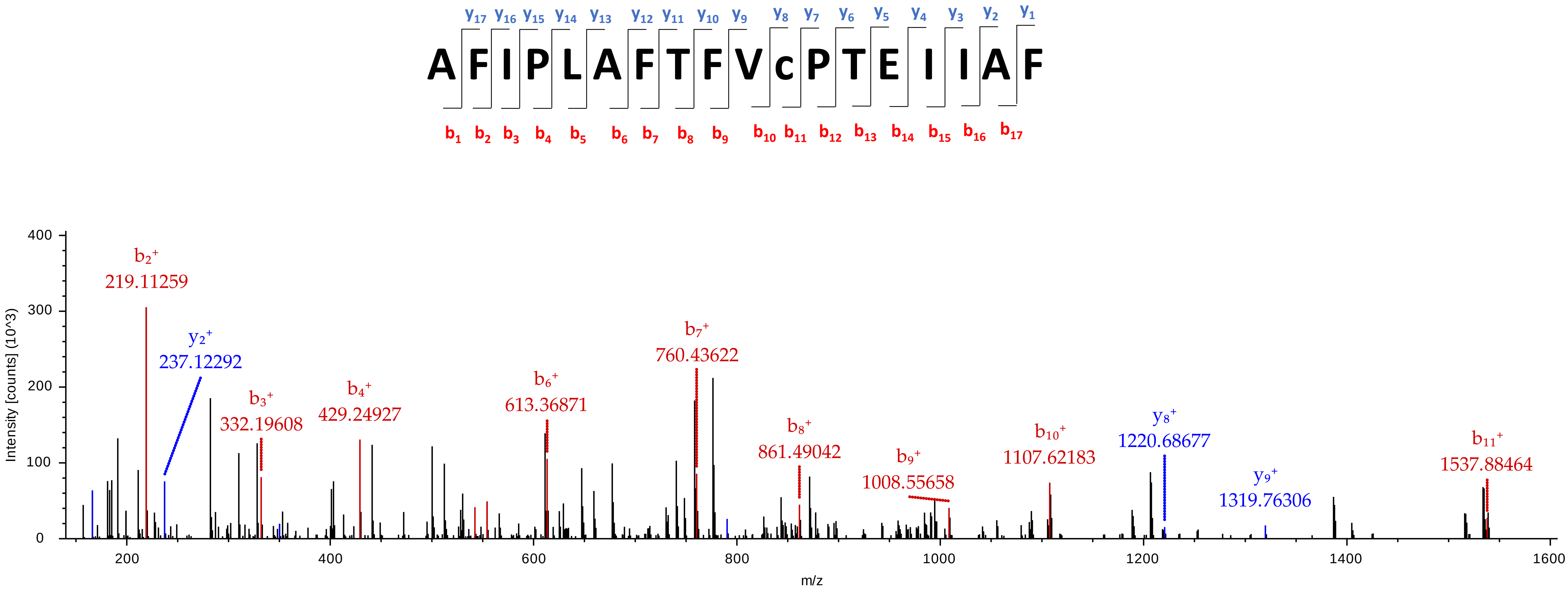

Supplementary Figure S5. MS/MS spectrum of the AFIPLAFTFVcPTEIIAF nitroalkylated peptide identified in the recombinant TSA1 protein treated with NO_2-OA. Fragmentation of the peptide precursor ion generated a series of peptide fragments identified as "b" if the charge was retained at the N-terminus or "y" if the charge remained at the C-terminus. Subscripts indicates the charge of ions.

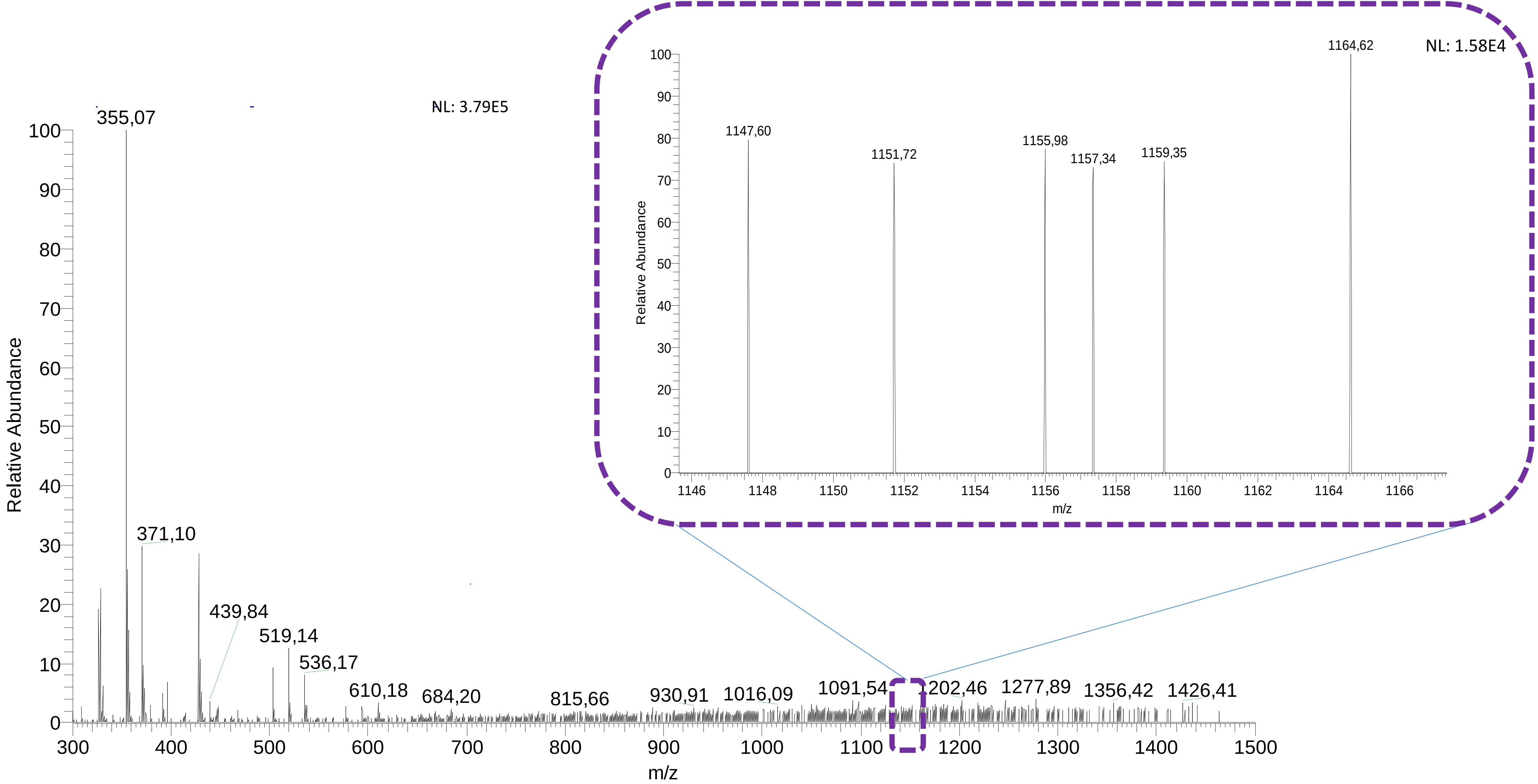

Supplementary Figure S6. Mass spectrum corresponding to the MS1 of the control sample where the m/z 1164.62 corresponding to the precursor ion of the AFIPLAFTFVcPTEIIAF peptide which contains the nitroalkylated Cys 47 from *in vivo* Tsa1 is shown. The intensity of the precursor ion was 1.58E4 and was localized at the RT 107.7 min. NL: normalization level.

Supplemental Table S1. Chromatographic and spectrometric characterization (experimental peptides, charge, molecular weight and RT) of the precursor ions that contain the targets susceptible to nitroalkylation detected in the recombinant Tsa1 treated with NO_2-OA. In the table, found the information of the unmodified peptides that contain the target susceptible to nitroalkylation as the nitroalkylated peptides of that target (in the nitroalkylated peptides the target appears in lowercase letters).

Target residue	Peptide experimentally	Chemical formula	Molecular weight (Da)	Charge	Retention time RT (min)	Window of RT (min)
Cys 47	VCPTEIIAF	C46H73N9O13S1	992,51208	+1	78,5	73-83
	TFVCPTEIIAF	C59H89N11O16S1	1240,63037	+2	79,7	75-85
	TFVcPTEIIAF	C77H122N12O20S1	1567,8674	+2	104,57	100-110
	AFIPLAFTFVcPTEIIAF	C118H179N19O27S1	2327,3013	+2	117,63	113-123
	AFTFVcPTEIIAF	C89H136N14O22S1	1785,9745	+2	107,61	103-113
Cys 170	TDKNGTVLPCNW	C58H90N16O19S1	1347,63457	+2	42,05	37-47
	TDKNGTVLPcNW	C76H123N17O23S1	1674,8759	+2	85,18	80-90
	TDKNGTVLPcNWTPGAATIKPTVEDSKEY	C155H248N36O51S1	3462,7828	+4	65,84	60-70
	QWTDKNGTVLPcNW	C92H141N21O26S1	1989,0121	+2	83,73	79-89
His 104	TNIPRKEGGLGPINIPLLADTNHSL	C116H194N34O36	2640,44472	+4	44,68	39-50
	LADTNHSLSRDY	C58H90N18O22	1391,65557	+2	21,59	16-27
	ADTNHSLSRDY	C52H79N17O21	1278,57085	+3	18,96	13-24
	LADTNhSLSRDY	C76H123N19O26	1718,8925	+3	61,88	56-66
	ADTNhSLSRDY	C70H112N18O25	1605,8095	+3	59,54	55-65
	LADTNhSL	C54H92N12O18	1197,6737	+2	66,52	61-71
His 135	IIDPKGVIRHITINDLPVGRNVDEAL	C127H215N37O38	2867,5997	+3	46,19	41-51
	IIDPKGVIRHITINDLPVGRNVDEALRL	C139H238N42O40	3136,7875	+5	47,04	42-52
	IIDPKGVIRHITINDLPVGRNVDEALRLVEAF	C161H268N46O46	3583,0099	+3	57,71	52-62
	IIDPKGVIRhITINDLPVGRNVDEAL	C145H248N38O42	3194,8492	+3	65,96	60-70

Supplementary Table S2. Characteristic m/z ratio and chromatographic retention time (RT) of peptides obtained form the nitroalkylation of recombinat Tsa1.

**Nitroalkylated target**	**Standard of Nitroalkylation: recombinant Tsa1 treated with NO_2-OA**		
	**Nitroalkylated peptide**	**m/z**	**RT**
**Cys 47**	TFVcPTEIIAF	784,9	104,57
	AFIPLAFTFVcPTEIIAF	1164,6	117,63
	AFTFVcPTEIIAF	893,9	107,6
**Cys 171**	TDKNGTVLPcNW	838,4	85,18
	TDKNGTVLPcNWTPGAATIKPTVEDSKEY	866,6	65,84
	QWTDKNGTVLPcNW	995,5	83,73
**His 105**	LADTNhSLSRDY	573,9	61,88
	ADTNhSLSRDY	536,2	59,54
	LADTNhSL	599,8	66,52
**His 136**	IIDPKGVIRhITINDLPVGRNVDEAL	1065,9	65,96
